# The Mediation and Moderation Effect Association among Physical Activity, Body-Fat Percentage, Blood Pressure, and Serum Lipids among Chinese Adults: Findings from the China Health and Nutrition Surveys in 2015

**DOI:** 10.3390/nu15143113

**Published:** 2023-07-12

**Authors:** Qinpei Zou, Chang Su, Wenwen Du, Yifei Ouyang, Huijun Wang, Bing Zhang, Shuquan Luo, Tao Tan, Yaokai Chen, Xiaoni Zhong, Huadong Zhang

**Affiliations:** 1 Chongqing Center for Disease Control and Prevention, Chongqing 400042, China; zouqinpei@163.com (Q.Z.); luosq2006@hotmail.com (S.L.); 2 School of Public Health, Research Center for Medicine and Social Development, Chongqing Medical University, Chongqing 400016, China; 3 Department of Infectious Diseases, Chongqing Public Health Medical Center, Chongqing 400036, China; yaokaichen@hotmail.com; 4 National Institute for Nutrition and Health, Chinese Center for Disease Control and Prevention, Beijing 100050, China; suchang@ninh.chinacdc.cn (C.S.); duww@ninh.chinacdc.cn (W.D.); ouyyf@ninh.chinacdc.cn (Y.O.); wanghj@ninh.chinacdc.cn (H.W.); zhangbing@chinacdc.cn (B.Z.); 5 Chongqing Health Statistics Information Center, Chongqing 401120, China; tantao1980@hotmail.com

**Keywords:** body-fat percentage, blood pressure, mediation effect, moderation effect, physical activity, serum lipids

## Abstract

Physical activity (PA) is of benefit and particularly important for cardiovascular disease risk factors as being sedentary becomes a lifestyle habit. Research into Chinese complex association among physical activity, body-fat percentage (BF%), blood pressure, and serum lipids is limited. The present study is based on an observational study among adults (>18 years old) residing in fifteen provinces in China. Data of 10,148 adult participants in the 2015 China Health and Nutrition Survey (CHNS) were analyzed. The simple mediation effect models with covariates were utilized to assess the association among PA and blood pressure or serum lipids, and BF% was played as a mediator. The serial multiple-mediator models with covariates were constructed to the further analysis of the relationship between PA and blood pressure, and BF% was the mediator 1 and blood lipids were the mediator 2. Based on the above hypothesis, the moderated mediation models with covariates were used to analyze the association among PA, BF%, and blood pressure; in addition, BF% was used as the mediator and blood lipids played as the moderator. In the simple mediation models, the model with a dependent variable was high-density lipoprotein cholesterol (HDL-C) or low-density lipoprotein cholesterol (LDL-C); BF% was played as the partly mediation effect and the proportion of contribution was 0.23 and 0.25, respectively. In the serial multiple-mediator models, blood lipids, as the second mediator, played the mediation effect; however, the effect was smaller than the BF%. In the moderated mediation model, blood lipids had the moderation effect as the moderator variable. HDL-C played a moderating role in the latter pathway of the “PA→BF%→SBP/DBP” mediation model, and LDL-C/TC played a moderating role in the direct effect of the “PA→BF%→DBP”. In conclusion, BF% played a mediating role in the relationship between PA and blood pressure. HDL-C, LDL-C, and TC were more likely to act as moderating variables in the mediation model “PA→BF%→SBP/DBP”. PA could directly and indirectly benefit to control the CVD risk factors simultaneously.

## 1. Introduction

More people die each year from cardiovascular diseases (CVD) than from any other cause. Hypertension, dyslipidemia, and obese are the independent risk factors of cardiovascular disease, and they often co-exist [1]. Hypertension—or elevated blood pressure (systolic blood pressure, SBP; diastolic blood pressure, DBP)—is a serious medical condition that significantly increases the risk of heart, brain, and other diseases. An estimated 1.4 billion people worldwide have high blood pressure, but just 14% have it under control [2]. In China, 23.2% of the Chinese adults aged 18 and above had hypertension in 2012–2015, as per the China Hypertension Survey [3]. Dyslipidemia—characterized by low high-density lipoprotein cholesterol (HDL-C), high-density lipoprotein cholesterol (LDL-C), high total cholesterol (TC), and high triglycerides (TG), has been recognized as an independent risk factor for atherosclerosis, coronary heart disease, and stroke. The latest report showed the prevalence of it among Chinese aged 18 years old and above, being 35.6% [4]. During the three national cross-sectional surveys undertaken from 2002 to 2015, serum lipids have increase sequentially among Chinese adults [5]. 

Obese is an independent chronic disease, with abnormal or excessive fat accumulation, which presents a risk to health. Its threats are CVD, diabetes, and even cancer [6]. In China, approximately half (overweight: 34.3%; obese: 16.4%) of adults were overweight or obese in 2018 [4]. Additionally, obese has the closed relationship with hypertension and dyslipidemia, but the underlying mechanism among them is not entirely clear. Most researchers hold the point that excess adipose tissue can cause systemic changes in the physiological and metabolic risk markers of these diseases [7]. Adipose tissue products are involved in the pathogenesis of essential hypertension [8]. As an endocrine organ, adipokines produced by adipocytes affect the transport and metabolism of lipids in blood [9], while changes in blood lipids affect blood pressure [10,11]. Body mass index (BMI) is regularly used as a robust measure of normal weight, overweight, and obese due to its simplicity and the modest correlation with adiposity [12]; and its use has been widely recommended in China [13]. However, it is a weak measure of individual fat mass, and healthy persons with high muscle mass can be misclassified as overweight or even obese [6]. Several studies have reported that high body-fat percentage (BF%) is an independent risk factor for cardiovascular diseases, coronary events [14,15,16,17], and all-cause mortality [18,19,20]. 

Physical activity (PA) refers to a person’s all movement, which has been shown to contribute to the prevention and management of CVD [21]. Previous studies have reported an inverse relationship between PA and BF% in middle-aged adults [22,23]. Also, international and domestic studies have reported the significant negative association between PA and the risk of hypertension [24,25,26,27,28], but also the opposite result [29]. A sedentary lifestyle with a lack of PA is the commonest secondary cause of dyslipidemia in developed nations [30]. However, current global estimates show one in four adults and 81.00% of adolescents do not perform enough PA recommended by the World Health Organization (WHO) [31]. In China, PA was observed to be in sharp decline as urbanization was dramatically increasing [32]. 

According to the previous studies and the results of our CHNS team, PA has beneficial effects on the chronic diseases above. However, the mechanism is not clear in relation to whether PA affects blood pressure and serum lipids through adipose, and the sequence of effects of PA on blood pressure and lipids has not been reported. Exploring the importance of body fat in the process of PA on CVD plays a vital role in lifestyle preventing CVD and improving health. Meanwhile, understanding the sequence of PA affects the risk factors of CVD has a positive effect on prevention and controlling the diseases at different course of diseases.

Mediation analysis is widely used in psychology and sociology to investigate the mechanism of interventions, but epidemiologists are also using it to analyze mechanisms in the “black box” in epidemiology in recent years; for example, exploring the mechanism of hand and knee osteoarthritis, frailty, disability, and other diseases [33,34,35,36]. However, studies in the mechanism of PA and CVD risk factors are limited in China. 

To address these knowledge gaps, data from Chinese adults (age ≥ 18 years) obtained in 2015 CHNS were used to examine the complex association among PA, BF%, blood pressure, and serum lipids. We hypothesize that the influence of PA on blood pressure and lipids partly comes from direct effect, and partly goes through BF% to effect on blood pressure and/or serum lipids. Specifically, we constructed the simple path “PA→BF%→SBP/DBP/HDL-C/LDL-C/TC/TG”. Based on the strengthened association between blood lipids and blood pressure, we constructed the serial mediators path “PA→BF%→HDL-C/LDL-C/TC/TG→SBP/DBP”. In addition, drawing from the interaction effect between BF% and serum lipids, we also constructed the more complex path based on “PA→BF%→SBP/DBP” as HDL-C/LDL-C/TC/TG played the role of moderator in it. According to these hypotheses, we aimed to assess the most reasonable path mechanism among PA, BF%, blood pressure, and serum lipids.

## 2. Materials and Methods

### 2.1. Study Population

We leveraged the 2015 wave of the CHNS, an ongoing multipurpose large-scale longitudinal survey, with the aim of capturing sociological, economic, and demographic factors that influence health and nutritional status across the Chinese life span. Until now, China has seen a total of 11 CHNS surveys (1989, 1991, 1993, 1997, 2000, 2004, 2006, 2009, 2011, 2015, and 2018) being conducted. During the thirty years, the survey provinces are increasing from eight provinces in 1989 to sixteen provinces in 2018. In 2015, there were fifteen provinces, including Shandong, Liaoning, Heilongjiang, Jiangsu, Henan, Guizhou, Hunan, Hubei, Zhejiang, Yunnan, Shanxi, and Guangxi and three autonomous cities (Beijing, Shanghai, and Chongqing). A multistage, stratified, random cluster sampling design was used to ensure a probability sample. Specific individuals participated in the survey repeatedly at each round unless they were lost to follow-up. Further details regarding the CHNS are described and can be accessed at the following internet website: https://www.cpc.unc.edu/projects/china (accessed on 1 March 2023), and elsewhere [37].

The 2015 survey included 7319 households within 360 communities (approximately 20 households per community). The number of participants (age ≥ 18 years) recorded who were not disabled, not pregnant, and not lactating was 16,799. Participants that omitted data on blood lipid profiles (*n* = 1859) or PA (*n* = 1742) or BF% (*n* = 335) were deleted.

**Figure 1:**
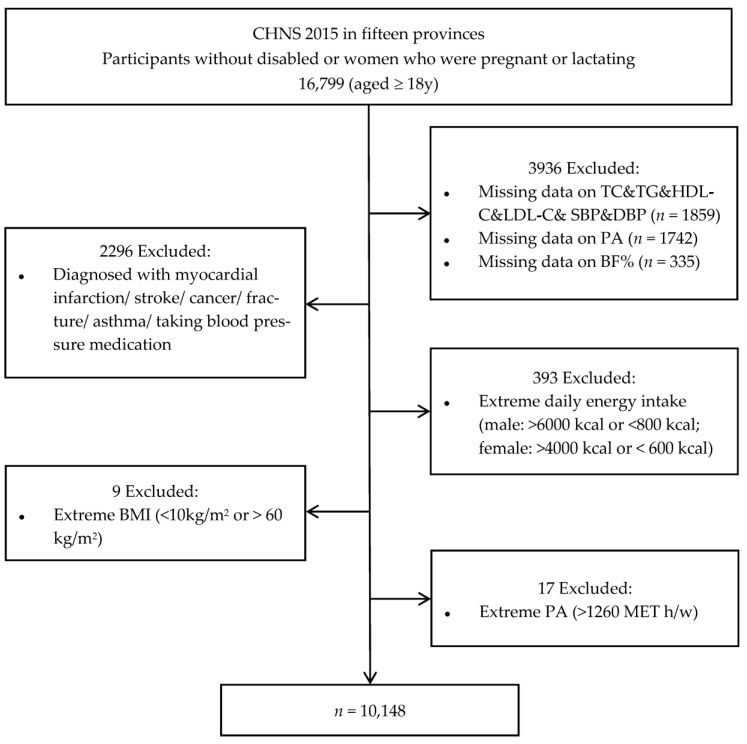
Flow chart of the included study participants from CHNS in 2015. TC, total cholesterol; TG, triglycerides; HDL-C, high-density lipoprotein cholesterol; LDL-C, low-density lipoprotein cholesterol; BMI, body mass index; PA, physical activity; BF%, body-fat percentage; SBP, systolic blood pressure; DBP, diastolic blood pressure.

After excluding participants that were associated with myocardial infarction, stroke, cancer, fracture, asthma, or taking blood pressure medication (*n* = 2296); and those associated with either extreme daily energy intake (higher than 6000 kcal/day or lower than 800 kcal/day for males, higher than 4000 kcal/day or lower than 600 kcal/day for females) (*n* = 393), those with extreme BMI (<10 kg/m^2^ or >60 kg/m^2^, *n* = 9), and those with extreme PA (>1260 metabolic equivalents of task hours per week (MET·h/week), *n* = 17), a total of 10,148 participants were included in the final analysis (Figure 1).

The survey protocols, instruments, and process of obtaining informed consent for this study were approved by the National Institute for Nutrition and Health, the Chinese Center for Disease Control and Prevention (No. 201524), and the Institutional Review Committee of the University of North Carolina at Chapel Hill; and written informed consent was obtained from all the CHNS participants before data collection.

### 2.2. Physical Activity and Sedentary Assessment

A standard PA questionnaire (which has been used in this ongoing cohort study for almost 30 years [38]) was relied on to calculate the average MET hours per week to indicate PA level. It included relevant questions on the intensity and time spent on four aspects, which included occupational, travel, leisure, and domestic movements. For example, farming belongs to occupational PA, biking to work is travel PA, playing basketball is leisure-time PA, and the food preparation is domestic movement. We defined a MET unit as the ratio of a person’s working metabolic rate relative to their resting (basal) metabolic rate. Then, based on the average intensity of each activity (or sub-activity) and the time spent on each activity, the measurements of the average MET hours per week were constructed. The Compendium of Physical Activities, based on the lowest level of detail available for each time-use survey [39,40,41], was used to estimate MET intensity values appropriately. The total MET hours per week was categorized into four quartiles, from the lowest to the highest PA level according to quartiles; i.e., Q1 (0~42.47 MET·h/week), Q2 (42.50~108.27 MET·h/week), Q3 (108.27~209.92 MET·h/week), and Q4 (209.93~1227.23 MET·h/week). Because there is not currently an internationally recommended standardized categorization for PA, details with respect to the calculation of these values have been previously described elsewhere [37,38].

Sedentary behaviors were calculated as the average hours per day (hour/day) spent in various non-occupational recreational activities, which was not included in the PA calculation. Lying down, sitting (reading/writing, playing board games, and using a computer or other forms of screen entertainment), and watching TV or movies/videos belong to sedentary behaviors. We used a summation of the time spent engaged in these activities to access the total time expenditure on sedentary behaviors.

### 2.3. Anthropometrics and Blood Biomarker Measurements

According to the standardized procedures, trained health workers or nurses measured the height, weight, body-fat percentage, waist circumference, and blood pressure of participants. Using a body composition analyzer (BC601, TANITA), weight was measured to the nearest 0.1 kg, with the participant in lightweight clothing standing without shoes. Body-fat percentage was calculated using the bioimpedance method utilizing a proprietary algorithm that requires age, sex, height, and PA level inputs by technicians. This method is being used widely and has previously been validated in other studies [42,43]. We recorded the total BF% which was the ratio of total body-fat mass and total body mass. We used the Seca 206 wall-mounted metal tapes to measure height without shoes to the nearest 0.1 cm. BMI was calculated as weight in kg divided by height in square meters. We used the Seca tape to measure waist circumference in centimeters at the mid-point between the lowest rib margin and the top of the iliac crest during light breathing. 

Blood pressure was obtained on the right arm, and all of the workers followed the standardized procedure using regularly calibrated mercury sphygmomanometers with appropriate-sized cuffs. We measured SBP at the first appearance of a pulse sound (Korotkoff Phase 1) and DBP at the disappearance of the pulse sound (Korotkoff Phase 5). Three measurements of SBP and DBP were averaged to reduce the effect of measurement error and the average of the three measurements was used in analysis. 

Overnight fasting blood was collected from the antecubital vein of participants in the morning. Serum and plasma samples were processed (centrifuged at 2000× *g* for 10 min at room temperature and separated into 9 aliquots) within two hours of collection in local hospitals. The aliquots were frozen and stored at −86 degrees centigrade for later laboratory analysis. All samples were analyzed in a national laboratory in Beijing with strict quality control. Serum TG, TC, HDL-C, and LDL-C were measured via the CHOD-PAP (Kyowa Medex Co., Ltd., Tokyo, Japan) method.

### 2.4. Assessment of Covariates

Standard questionnaires were used by well-trained interviewers to collect the sociodemographic data, including sex, age, current smoking status, alcohol consumption, and annual per capita household income. Educational level was divided into three categories; i.e., primary school education and illiteracy, junior school education, and high school education and above. We defined marital status as “married” and “not married”. Participants reported their gross annual per capita household income according to household size, and their 2009 gross annual income was inflated to year 2015 equivalency. We calculated the energy intake, energy from dietary fat, dietary cholesterol, and dietary sodium-to-potassium ratio, which were collected using three consecutive 24 h recalls (two weekdays and one weekend), in combination with the household weighing of condiments over the same three-day period, according to the Chinese Food Composition Tables. 

### 2.5. Statistical Analysis

The mediation effect model [44] is a statistical method used to evaluate evidence from studies designed to test hypotheses about how some causal antecedent variable *X* transmits its effect on a consequent variable *Y*. Mediator is the link connecting the relationship between *X* and *Y*, which theoretically means some internal mechanism. They represent a possible or proposed mechanism—the contents of the “black box”—by which message framing influences behavior. Once *X* exerts its effect on *M*, then *M*’s causal influence on *Y* produces a variation in *Y*. Based on ordinary least-square regression, the mediation effect model can explain the mechanism of path analysis and demonstrate how independent variables affect dependent variables when they are divided into direct effects and indirect effects.

#### 2.5.1. The Simple Mediation Effect Models

**Figure A1:**
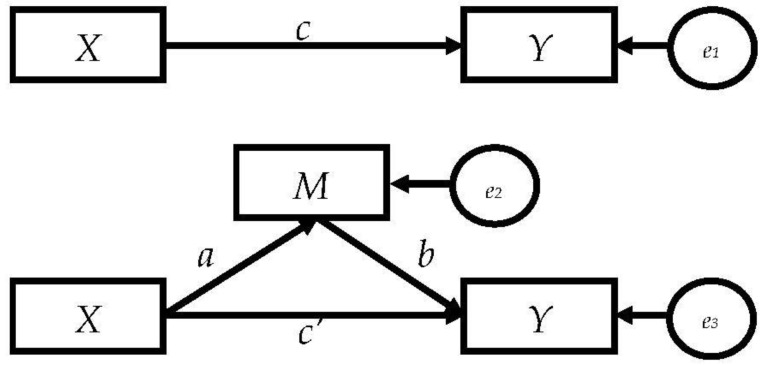
A conceptual diagram of a simple mediation model.

The most basic of mediation models—the simple mediation model—is represented in conceptual diagram form in Figure A1. It is any causal system in which at least one causal antecedent *X* variable is proposed as influencing an outcome *Y* through a single intervening variable *M*. There are two pathways by which *X* can influence *Y* in this model. These pathways are found by tracing the ways one can go from *X* to *Y* while never tracing in a direction opposite to the direction an arrow points. One pathway leads from *X* to *Y* without passing through *M* and is called the direct effect of *X* on *Y*. The second pathway from *X* to *Y* is the indirect effect of *X* on *Y* through *M*. The indirect effect represent show *Y* is influenced by *X* through a causal sequence in which *X* influences *M*, which in turn influences *Y*.

In Figure A1, *a*, *b*, and *c*′ are the regression coefficients given to the *X* in the model in the estimation of the consequents; and *e* is the error. In this diagram, *c*′ estimates the direct effect of *X* on *Y.* The indirect effect of *X* on *Y* through *M* is the product of *a* and *b*. The indirect effect tells us that two cases that differ by one unit on *X* are estimated to differ by *ab* units on Y as a result of the effect of *X* on *M* which, in turn, affects *Y*. The direct and indirect effects perfectly partition how differences in *X* map on to differences in *Y*; the total effect of *X* and denoted here as *c*. This relationship can be written as *c* = *ab* + *c*′. 

About the estimating coefficients, the most efficient power of test is Bootstrap in mediation effect analysis as mediation model developing. It uses the repeated sampling method to test the product of test coefficients (e.g., *ab*), and this test process is also the core of the mediation effect. 

To judge the mediation effect, there was a procedures of mediation effect testing according to the Wen’ theory [45]. The flow chart of the mediation effect testing is listed in Figure A7. 

**Figure A2:**
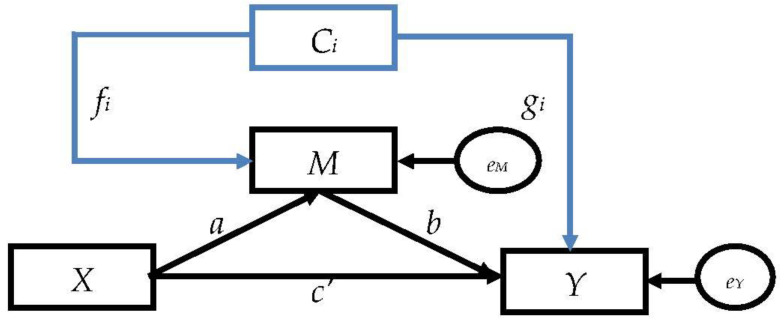
A conceptual diagram of a simple mediation model with covariate.

In fact, we added the covariates in mediation models, e.g., age, current smoking status, alcohol consumption, annual per capita household income, to control the effect on outcome *Y.* Then, we conducted the mediation model with covariates. *C_i_* represents the covariates, and *f_i_* and *g_i_* were the coefficient of *C_i_* on *M* and *Y*, respectively, in Figure A2.

#### 2.5.2. The Serial Multiple-Mediator Models 

**Figure A3:**
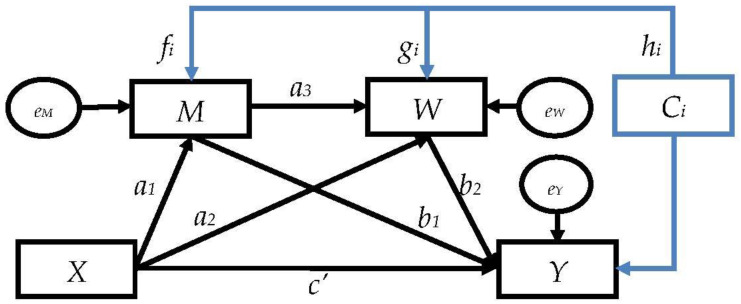
A conceptual diagram of a serial multiple-mediator model with covariate.

The goal when an investigator estimates a serial multiple-mediator model is to investigate the direct and indirect effects of *X* on *Y* while modeling a process in which *X* causes *M*, which in turn causes *W*, and so forth, concluding with *Y* as the final consequent (Figure A3). X is modeled as affecting Y through four pathways. One pathway is indirect and runs from *X* to *Y* through *M* only, a second indirect path runs through *W* only, and a third indirect influence passes through both *M* and *W* in serial, with *M* affecting *W*. The remaining effect of *X* is direct from *X* to *Y* without passing through either *M* or *W*.

The total effect of *X* on *Y* partitions into direct and indirect components, just as it does in the simple mediation model. This model has three specific indirect effects and one direct effect. The three specific indirect effects are each estimated as the product of the regression weights linking *X* to *Y* through at least one mediator.

The specific indirect effect of *X* on *Y* through only *M* is *a*_1_*b*_1_, the specific indirect effect through *W* only is *a*_2_*b*_2_, and the specific indirect effect through both *M* and *W* in serial is *a*_1_*a*_3_*b*_2_. When the total indirect effect of *X* is added to the direct effect of *X*, the result is *c*, the total effect of *X*, which can be estimated from a regression estimating *Y* from *X* only; the equation is *c = c*′ *+ a*_1_*b*_1_ + *a*_2_*b*_2_ + *a*_1_*a*_3_*b*_2_. 

*C_i_* is also represents the covariates; and *f_i_*, *g_i_*, and *h_i_* were the coefficients of *C_i_* on *M*, *W*, and *Y*, respectively, in Figure A3.

Compared with the simple mediation model, the serial multiple-mediator models could obtain the total mediation effect. Meanwhile, under the premise of controlling other mediation variables (such as M), the specific mediation effect of each mediation variable (such as W) is focused. It could reduce the parameter estimation deviation caused by ignoring other mediators in the simple mediation model. It also could compare the importance degree of mediation effects among several mediators. We can analyze the model from three aspects. Firstly, there are three specific pathways. Path 1 is “*X*→*M*→*Y*”, and the product of the test coefficient is *a*_1_*b*_1_. Path 2 is “*X*→*W*→*Y*”, and the product of the test coefficient is *a*_2_*b*_2_. Path 3 is “*X*→*M*→*W*→*Y*”, and the product of the test coefficient is *a*_1_*a*_3_*b*_2_. Secondly, the total mediation effect is *c*, *c = c*′ *+ a*_1_*b*_1_ + *a*_2_*b*_2_ + *a*_1_*a*_3_*b*_2_. Thirdly, we compared the mediation effect (|*a*_1_*b*_1_ *− a*_2_*b*_2_*|*, |*a*_1_*b*_1_ *− a*_1_*a*_3_*b*_2_|, and |*a*_2_*b*_2_ *− a*_1_*a*_3_*b*_2_|).

#### 2.5.3. The Moderated Mediation Models

**Figure A4:**
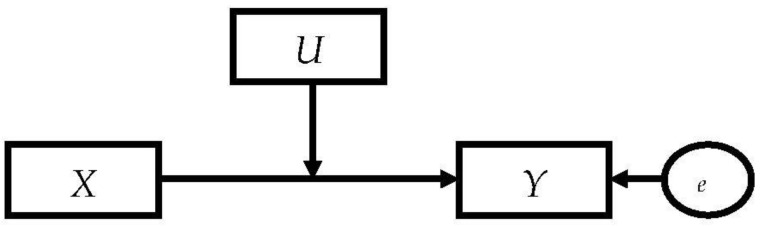
A conceptual diagram of a simple moderation model.

Moderation is depicted in the form of a conceptual diagram in Figure A4. This diagram represents a process in which the effect of *X* on *Y* is influenced by or dependent on *U*, as reflected by the arrow pointing from *U* to the line from *X* to *Y*. Then, *U* affects the direction (positive and negative) and strength. 

**Figure A5:**
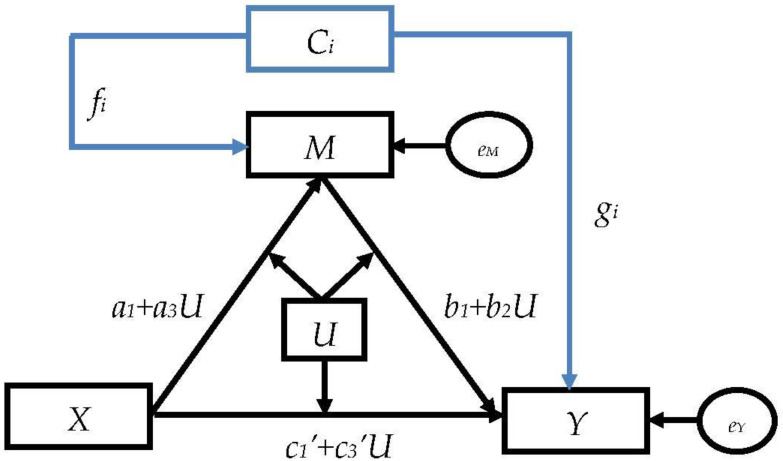
A conceptual diagram of a moderated mediation model with covariates.

**Figure A6:**
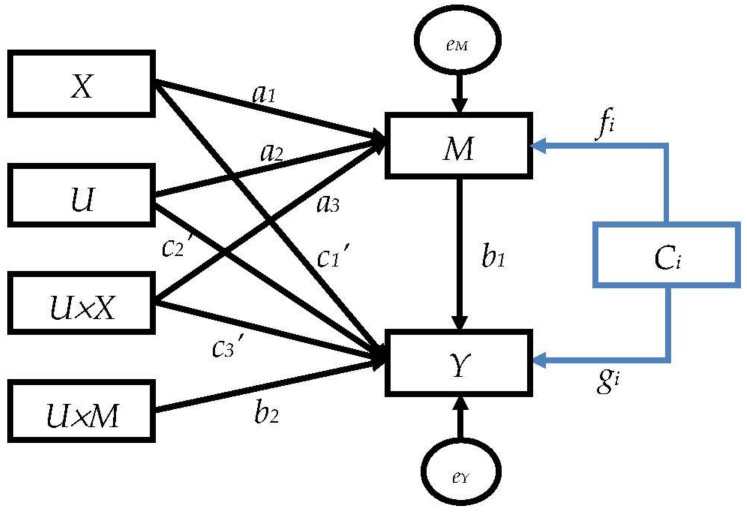
A statistical diagram of a moderated mediation model with covariates.

**Figure A7:**
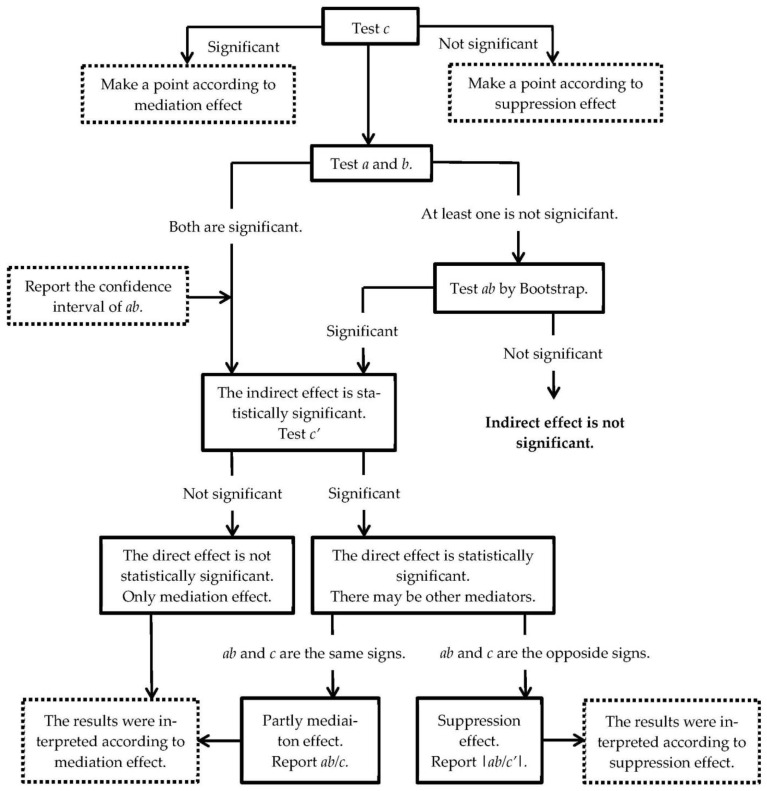
A flow chart of the mediation effect testing.

According to the theoretical assumption and model comparison, we conducted the moderated mediation model with covariates. Figure A5 is the conceptual diagram of a moderated mediation model with covariates, and Figure A6 is the statistical diagram of it. It can be seen that *a*_1_ is the effect of *X* on mediator *M*, *a*_2_ is the effect of moderator *U* on *M*, and *a*_2_ is the effect of the interaction effect between *X* and *U* on *M*. Coefficient *b*_1_ is the effect of *M* on *Y* controlling *X*, *U*, and covariate *C_i_*; and *b*_2_ is the effect of the interaction effect between *U* and *M* on *Y*. Coefficient *c*_1_′ is the effect of *X* on *Y* controlling *M*, *U*, and *C_i_*. Coefficient *c*_2_′ is the direct effect of *U* on *Y* controlling *X* and *M*. Coefficient *c*_3_′ is the effect of the interaction effect between *U* and *X* on *Y*. Coefficients *f_i_* and *g_i_* are the effect of *C_i_* on *M* and *Y*, respectively.

Whether the moderator *U* has a moderation effect on the mediator *M* and the dependent variable *Y* is determined by testing whether *a*_3_, *b*_2_, and *c*_3_′ are statistically significant. Coefficient *a*_3_ is statistically significant, which indicates that *U* has a moderation effect on the influence of *X* on *M*. Coefficient *b*_2_ is statistically significant, which indicates that *U* has a moderation effect on the influence of *M* on *Y*. Coefficient *c*_3_′ is statistically significant, which indicates that *U* has a moderation effect on the influence of *X* on *Y*.

Through a Bootstrap test on the moderated mediation effect (*a*_1_ + *a*_3_*U*)(*b*_1_ + *b*_2_*U*), the report gives the results of 95%*CI* at the 16th, 50th, and 84th percentiles. If the 95%*CI* excludes zero, then the moderated mediation effect is statistically significant. Otherwise, the effect is not statistically significant.

All the equations and other details of mediation models above were omitted because of the limited space, and they could be found in previous studies [44].

**Figure A8:**
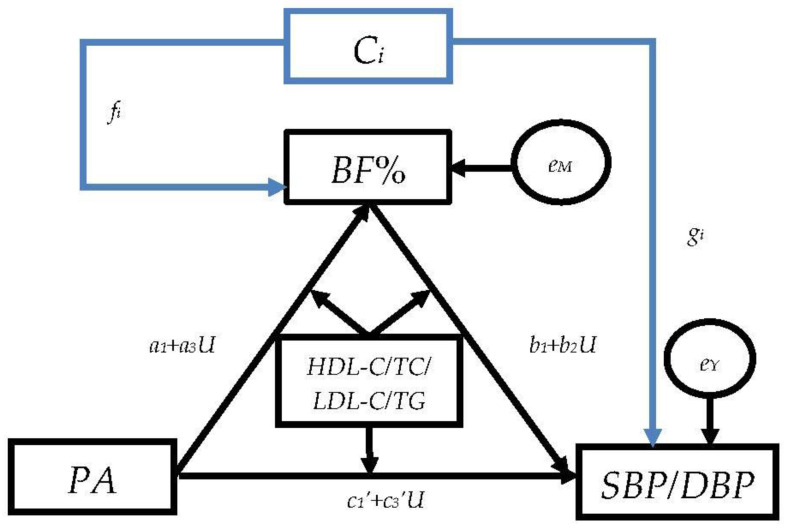
A specific conceptual diagram of a moderated mediation model with covariates.

We constructed three models according to the theoretical hypothesis. Mode 1 is the simple mediation model with covariates. “PA→BF%→SBP/DBP/HDL-C/LDL-C/TC/TG”. BF% is the mediator. Model 2 is the serial multiple-mediator model with covariates. “PA→BF%→HDL-C/LDL-C/TC/TG→SBP/DBP”. BF% is the mediator *M* and blood lipids are the mediator *W*. Model 3 is the moderated mediation model with covariates. “PA→BF%→SBP/DBP”. BF% is the mediator, blood lipids are the moderator *U* (Figure A8). The conceptual diagrams of the hypothesis are clear. The samples are sufficient. The Bootstrap method is used for repeated sampling 5000 times, and the interval estimation is given, which accords with the application conditions of the mediation effect model.

We calculated descriptive statistics for the individual demographic variables in 2015 and stratified them by sex. The median and the 25th and 75th percentiles were used to express continuous variables because of their abnormal distribution. Categorical variables were expressed as number (percentage). 

All statistical descriptions were conducted using SAS software (version 9.4, SAS Institute Incorporated, Cary, NC, USA). The mediating and moderating model used IBM SPSS Statistics 25 and the plug-in PROCESS by Hayes’ team [44]. We selected model 4, model 6, and model 59 to conduct the mediation and moderation model analysis. All the continuous variables were centrally processed using (x − $\bar{x}$). A *p*-value of <0.05 was considered to be statistically significant.

## 3. Results

### 3.1. Basic Characteristics of Participants in 2015

Table 1 shows the primary information of participants for each sex and the total samples in 2015. There were 10,148 sample participants in 2015, and the percentage of male participants was 45.79%. The median age was 50.57 years old. The number of participants with high school education and above was 36.14%, which was the highest percentage of education types. The annual per capita household income was 16,100 yuan. The percentages of current smokers and alcohol drinkers were 24.06% and 28.65%, respectively. The median values of energy intake, energy from dietary fat, dietary cholesterol, and dietary sodium-to-potassium ratio were 1930 kcal/day, 35.34%, 207.21 mg/d, and 2.67, respectively. The median of PA and sedentary activity time were 108.27 MET·h/week and 16.00 h/week, respectively. The median of BF% was 28.00%, and that of females was higher than that of males. The median values of SBP, DBP, HDL-C, LDL-C, TG, and TC were 122.00 mm Hg, 80 mm Hg, 1.25 mmol/L, 3.02 mmol/L, 1.18 mmol/L, and 4.83 mmol/L, respectively.

### 3.2. The Simple Mediation Models

Based on the simple mediation models with concomitant variables, we constructed the path “PA-BF%-blood pressure/ serum lipids”. Table 2 presents the coefficients (95%*CI*) of the simple mediation models between PA and blood pressure, serum lipids among Chinese adults from fifteen provinces in 2015; in addition, Figure S1 illustrates the path diagram of the models and the coefficient of each path. The coefficients of the indirect effect between PA and SBP/DBP were statistically significant, but the total effects were not. Furthermore, the coefficients of the direct effect of SBP were not statistically significant and they were called the mediation effect. On the other hand, the coefficients of the direct effect of DBP were statistically significant, but the symbols of direct effect and indirect effect were opposite; they were known as the suppression effect.

The total effect coefficient between PA and HDL-C was 0.0229, and the direct effect and the indirect effect were 0.0177 and 0.0052, respectively. They were all statistically significant. This meant the indirect effect occupied the total effect 0.23, and it was called the partly mediation effect. The situation of LDL-C was similar to HDL-C; the ratio of indirect effect and direct effect was 0.25. However, the association between PA and TC/TG/SBP belonged to the mediation effect because the coefficient of direct effect was not statistically significant. Due to the opposite signs about the coefficients of direct and indirect effect, BF% played the suppression effect, and the ratio between *ab* and *c’* was 0.48.

### 3.3. The Serial Two Mediator Models

According to the results from Table 2, we observed the direct and indirect effect from PA to blood lipids. The previous studies provide a strong relationship between serum lipids and blood pressure. Then, we further considered that blood lipids as another mediator played the mediating role in the effects of PA on SBP and DBP. The two mediators’ mediating models were constructed.

Table 3 illustrates that in the association between PA and SBP/DBP, when one finds BF% as the mediator M and blood lipids as the mediator W, all the coefficients of total and direct effects in the hypothetical paths were not statistically significant; however, the coefficients of total mediation effects were significant. 

Path 1 “PA→BF%→SBP/DBP” was established, which was consistent with the results in Table 2. However, in path 2, only “PA→HDL-C/LDL-C→SBP” and “PA→LDL-C→DBP” were established. These meant that HDL-C and LDL-C had the mediation effect in the effect from PA on SBP/DBP. We observed that in the path “PA→HDL-C→SBP/DBP”, the coefficients of *a*_2_*b*_2_ were positive, which were contrary to the common sense. Then, we drew mediation effect path diagrams to carefully analyze the reasons (Figure S2). Figure S2A shows the coefficient *b*_2_ between HDL-C and SBP was 1.4097 (*p* < 0.05), and Figure S2E shows the coefficient *b*_2_ between HDL-C and DBP was 0.1289 (*p* > 0.05). These meant SBP would increase as HDL-C was increasing. Therefore, the coefficients *a*_2_*b*_2_ in path 2 were positive in Figure S2A,E, but the coefficient in Figure S2E was not statistically significant. In Figure S2B,F, the association between LDL-C and SBP/DBP was positive, which meant the blood pressure would increase as HDL-C/LDL-C was increasing. In Table 3, the coefficients *a*_2_*b*_2_ in path 2 were not statistically significant in the Bootstrap tests when TC or TG played as the second mediator *W*.

In path 3, except the path “PA→BF%→HDL-C→DBP”, the coefficients *a*_1_*a*_3_*b*_2_ in other paths “PA→BF%→HDL-C/LDL-C/TC/TG→SBP/DBP” were statistically significant in the Bootstrap tests. We hold the opinion that BF% and blood lipids acted as the mediators simultaneously in the hypothesis models, and they were also showed sequential effect. The contrast mediation effect was reflected by the difference value of the regression coefficients of path 1, path 2, and path 3. In the paths “PA→BF%→HDL-C→SBP/DBP”, the coefficients in path 1 were negative, and the coefficients in path 2 and path 3 were positive. The opposite signs indicate that the effect in path 2 and path 3 had the opposite effect, called the inhibitory effect. In all the paths, |*a*_1_*b*_1_*|* > |*a*_2_*b*_2_| > |*a*_1_*a*_3_*b*_2_|, which meant that BF% played a greater mediation role than blood lipids among all the mediation effects.

### 3.4. The Moderated Mediation Models

In view of the fact that the mediation effects of serum lipids were not as important as BF% in the serial multiple-mediator models, especially in the path 2 in Table 3, only the mediation effects of HDL-C and LDL-C were statistically significant. Therefore, we constructed the moderated mediation models, in which blood lipids played as the moderator in the front path (PA→BF%), back path (BF%→SBP/DBP), and the direct path (PA→SBP/DBP) in the models “PA→BF%→SBP/DBP” (Figure A8).

**Figure 2:**
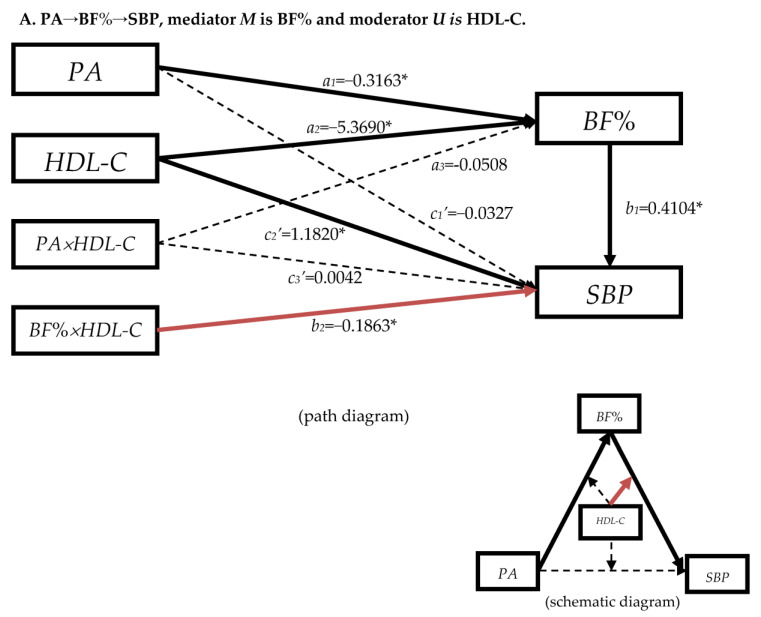

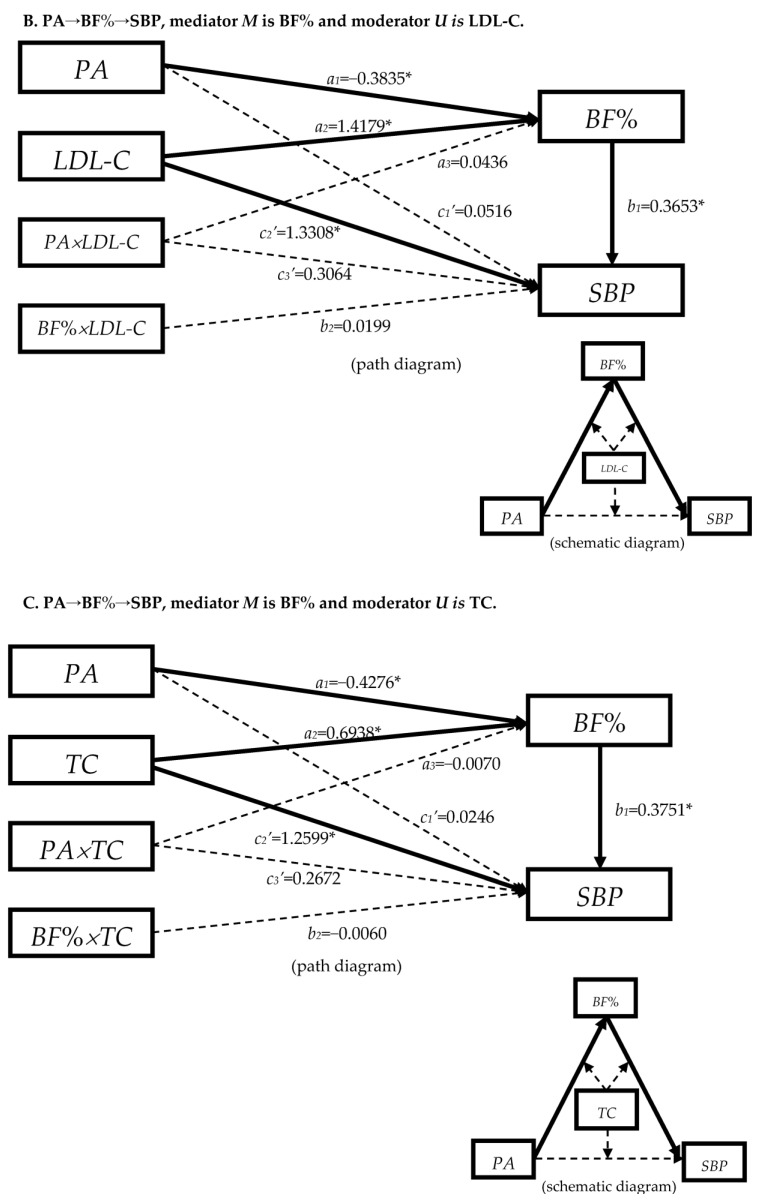

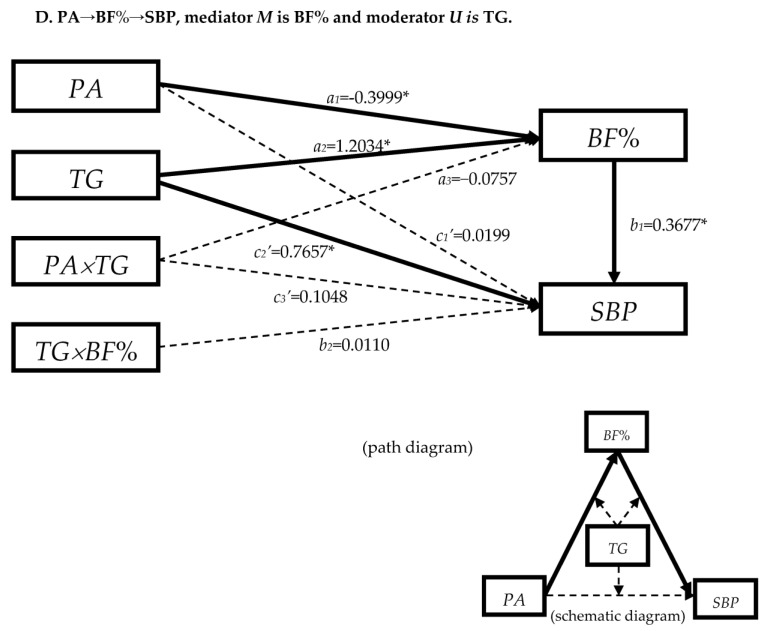
Diagram of the moderated mediation models between physical activity and SBP among Chinese adults from fifteen provinces in 2015. The mediator is the body-fat %. The moderators are blood lipids. The arrows indicate the direction. The solid line indicates that the coefficient is statistically significant and the pathway is established. The dashed line indicates that the coefficient is not statistically significant and the pathway is not valid. Red arrow means the moderation is statistically significant. Omit the covariates and error terms. * *p* < 0.05. The moderator *U* for A, B, C, and D is HDL-C, LDL-C, TC, and TG, separately.

**Figure 3:**
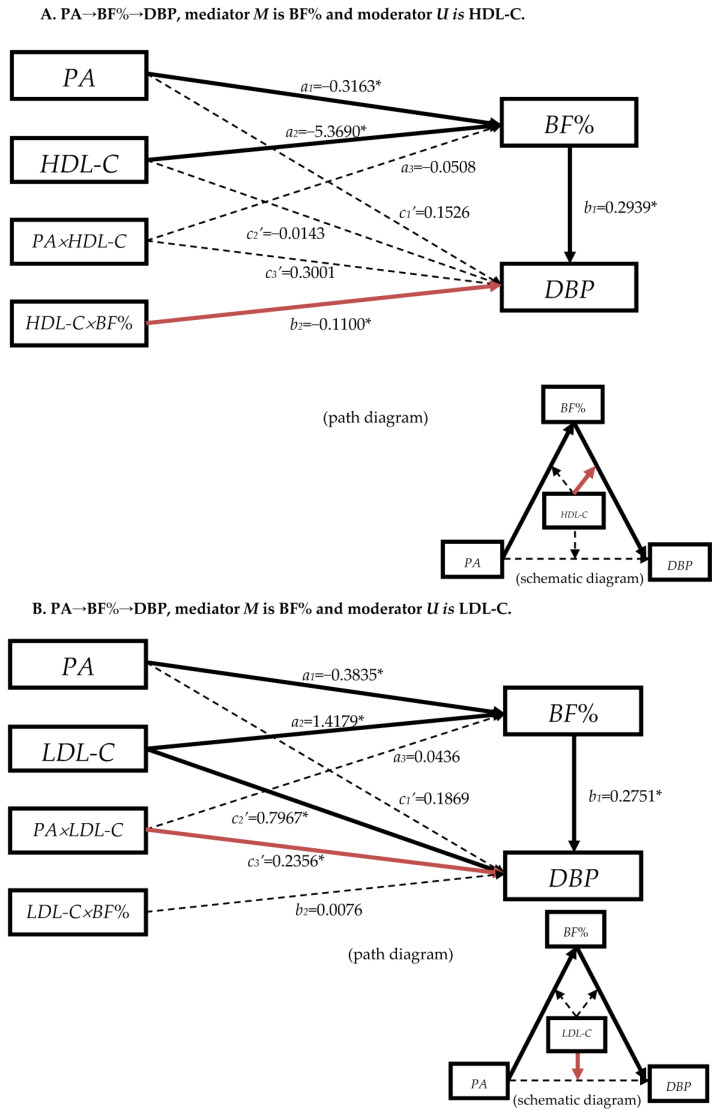

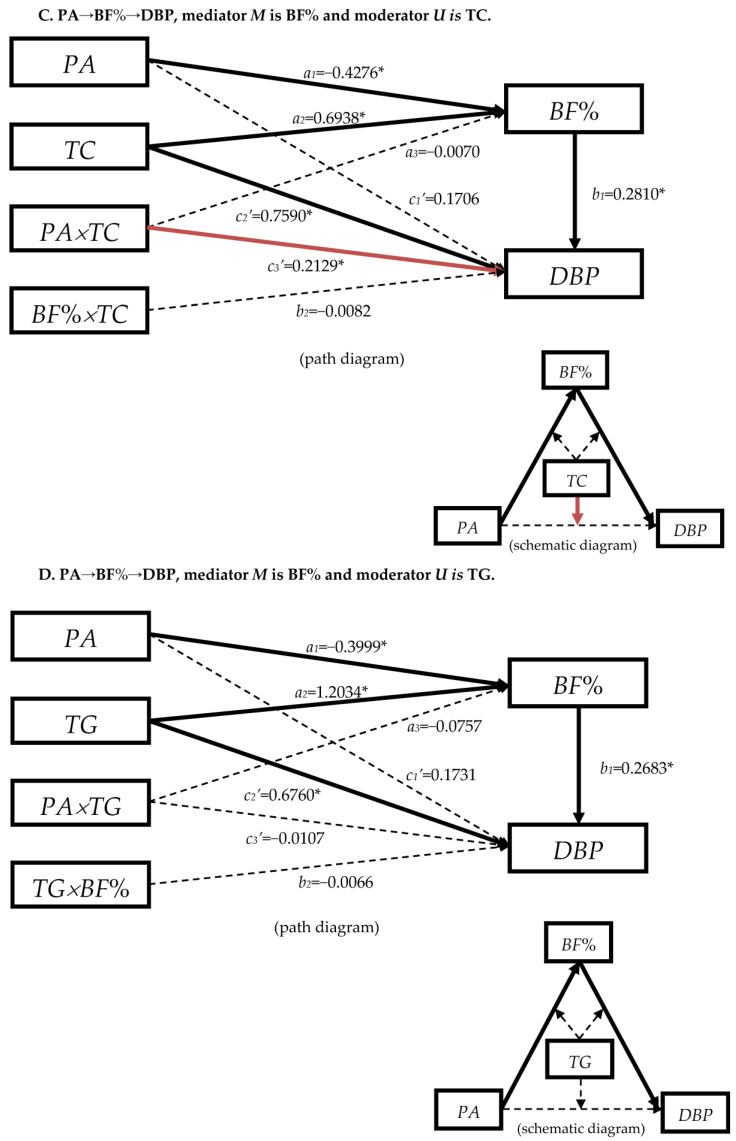
Diagram of the moderated mediation models between physical activity and DBP among Chinese adults from fifteen provinces in 2015. The mediator is the body-fat %. The moderators are blood lipids. The arrows indicate the direction. The solid line indicates that the coefficient is statistically significant and the pathway is established. The dashed line indicates that the coefficient is not statistically significant and the pathway is not valid. Red arrow means the moderation is statistically significant. Omit the covariates and error terms. * *p* < 0.05. The moderator *U* for A, B, C, and D is HDL-C, LDL-C, TC, and TG, separately.

In Figure 2 and Figure 3, the mediation effects of paths ”PA→BF%→SBP/DBP” were established, which were consistent with the results of simple mediation effect models (Table 2). When blood lipids indexes were played as moderators, they effected the BF% and blood pressure directly, but the moderate effects were different in different paths.

#### 3.4.1. The Moderation Effect of Blood Lipids in Mediation Model “PA→BF%→SBP”

In Figure 2A, the coefficient between HDL-C and BF% was −5.3690 (*p* < 0.05), and the value between HDL-C and SBP was 1.1820 (*p* < 0.05), which meant that BF% would decrease while SBP would increase when HDL-C was increasing. The coefficients between PA × HDL-C and BF%/SBP were not statistically significant (dashed line), which meant HDL-C had no moderation effect in the front path of the mediation effect and the direct effect. However, HDL-C showed the moderation effect in the back path of the mediation effect because of the statistically significant coefficient (*b*_2_ *=* −0.1863, *p* < 0.05) between BF% × HDL-C and SBP (the red solid arrow). 

In Figure S2A, HDL-C was played as the second mediator, which had the mediation effect in the path between BF% and SBP. However, HDL-C had the moderation effect in the path between BF% and SBP in Figure 2A. These indicated that HDL-C affected SBP after BF%; meanwhile, it also acted back to BF% and, together with BF%, it moderated SBP. 

In Figure 2B–D, LDL-C, TC, and TG had no moderation effect in the path with the present conditions of the models as the arrows “BF% × HDL-C→SBP” were black dashed. But, Figure S2B–D provided the evidence that, as the mediators, they had the mediation effect on SBP after BF%.

#### 3.4.2. The Moderation Effect of Blood Lipids in Mediation Model “PA→BF%→DBP”

In Figure 3A, HDL-C had the moderation effect at the back-path (*b*_2_ *=* −0.1100, *p* < 0.05). It would not affect DBP directly (*c*_2_′ = −0.0143, *p* > 0.05), but moderated DBP together with BF% (the red solid arrow). The result was consistent with Figure S2E (*b*_2_ = 0.1289, *p* > 0.05).

Figure 3B shows LDL-C had the moderation effect in the direct effect from PA to DBP (*c*_3_′ = 0.2356, *p* < 0.05, the red solid arrow). This moderated direct effect was statistically significant at the 84th percentile (*β* = 0.3866, *p* = 0.0040; it was not marked in the Figure 3B). LDL-C would not moderated BF% and DBP as the coefficients were not statistically significant (*c*_2_′ *=* 0.7967, *b*_2_ *=* 0.0076, *p* > 0.05). TC as the moderator had a similar effect to LDL-C in the path “PA→BF%→DBP”, as Figure 3C indicates. It also had the moderation effect in the direct effect from PA to DBP (*c*_3_′ = 0.2129, *p* < 0.05, the red solid arrow); and the moderated direct effect was statistically significant at the 84th percentile (*β* = 0.3704, *p* = 0.0043, it was not marked in the Figure 3C). 

Figure 3D illustrates that TG had no moderating effect in any path with the present conditions of the models as the arrows “BF% × TG→DBP” were black dashed in the path diagram.

As a brief summary, HDL-C moderated the back path of the mediation model “PA→BF%→SBP/DBP”, while TG had no moderated effect in these two models. LDL-C and TC moderated the direct effect “PA→DBP” rather than “PA→SBP”.

## 4. Discussion

This large-scale survey among participants who were ≥18 years old provides comprehensive estimates of the association among PA, BF%, blood pressure, and serum lipids in China. According to our hypotheses, in the simple mediation effect, BF% played the mediation effect of PA on TC and TG, but the partly mediation effect on HDL-C and LDL-C. Meanwhile, BF% played the mediation effect of PA on SBP, but the suppression effect on DBP. In addition, the moderation effect of blood lipids was observed in the mediation model “PA→BF%→SBP/DBP”. These succinctly explain the association among PA and the main CVD risk factors among Chinese adults; in addition, they focus on the internal relationship when PA and outcome variables did not show the direct relationship, and explore the possible reasons through the mediation effect analysis.

In the simple mediation effect models, BF% played the partly mediation effect on HDL-C and LDL-C when adjusting the confounders, and the proportion of this part accounted for 23% and 25% of the total effect, respectively. It also indicated that the direct effect, PA on HDL-C or LDL-C, accounted for the main effect. Figure S1c showed that PA had a positive effect on HDL-C, while BF% had a negative effect on HDL-C [7,10]; then, the increasing PA could decrease BF% and increase HDL-C at the same time, and the decreasing BF% could also help to increase HDL-C. PA had a negative effect on LDL-C, while BF% had a positive effect on LDL-C, which meant, in the two paths, that PA-decreased LDL-Cs were established. When the PA was increasing, both BF% and LDL-C were decreasing, and the less adipose tissue would lead to a lower level of LDL-C to some extent. Based on the partly mediation effect when BF% was the mediator in the model of PA on HDL-C/LDL-C, even if the direct effect was statistically significant, the other mediators might have existed, such as insulin sensitivity. Accordingly, we should conduct further analysis.

In the hypothesized model, PA improved TC and TG through the mediation effect of BF%, but the direct effects (Figure S1e,f). The previous studies showed that there was a positive association between body fat and TG [7], and it was consistent with our results. However, Yang reported a zero association between BF% and TC [10], which was different from our results. Su et al. found that dietary cholesterol had a certain effect on serum TC [46], while our results showed a statistically significant positive association between BF% and TC under the control of dietary cholesterol. Therefore, considering the special population of Yang’s study, overweight and obese adults, it was reasonable to attribute the different results between BF% and TC to the different sample population; ours consisted of adults aged 18 years and above. 

Body fat is generally thought to affect lipid metabolism through fat accumulation and distribution or through different signaling pathways. Some scholars also believed that adipose tissue affects lipid metabolism by releasing various adipokines. These adipokines exert their biological effects through different chemical transmitters and signaling pathways and have dual effects on the cardiovascular system. For example, adipokines, such as leptin, resistin, and chemoattractant, have the effect of promoting atherosclerosis and endothelial dysfunction, while adiponectin is a strong anti-inflammatory factor with anti-atherosclerotic effects; in addition, omentin and visfatin are positively correlated with HDL-C [47]. It can be inferred from our results that PA increases the energy expenditure of the body and reduces the size of adipocytes; it also induces adipose tissue to release more beneficial adipokines into the blood and inhibits the release of harmful adipokines, which regulates lipid metabolism pathways and improves dyslipidemia.

The hypothesized pathway “PA→BF%→HDL-C/LDL-C/TC/TG” in this study was valid in a population-based observational study, indicating that PA has a dual effect on adipose tissue content and releasing adipokines. In terms of improving blood lipids, BF% has a greater regulatory effect on TG than HDL-C, LDL-C, and TC because the absolute value of β was larger than the other three (Figure S1f). However, the complex regulatory mechanism still needs to be verified by experiments at the cellular and molecular levels. Our results suggest that adipose tissue lowering is an important way to prevent and control dyslipidemia, and increasing PA can affect body fat and blood lipid at the same time.

BF% played the mediation effect of PA on blood pressure, and serum lipids tended to be the moderator in the hypothesized models. According to our previous [6] and the present study, there was a statistically significant negative association between PA and BF% in both sexes. When PA was elevating, BF% was dropping. BF% increasing was often accompanied by an increase in blood pressure. Yang [10] reported that their team measured a sample of 1322 Chinese with overweight or obese aged 20 to 55 years old using a DXA method, and analyzed the relationship between BF% and blood pressure. They found BF% in both males and females was closely associated with SBP and DBP (*β*_SBP,male_ = 0.21, *β*_DBP,male_ = 0.21, *β*_SBP,female_ = 0.19, *β*_DBP,female_ = 0.21) after adjusting the age. However, only one confounder in the model was not reasonable, and it was necessary to control the factors that had an important influence on blood pressure, such as alcohol consumption and dietary sodium-potassium ratio. Hariri [7] reported that the skewness coefficients between body-fat mass and SBP/DBP by the DXA method were 0.22 and 0.27, respectively. The results of the above literatures indicated that BF% was positively correlated with blood pressure, but the strength of the relationship was not high enough under certain conditions of controlling confounding factors.

The epidemiology evidence showed that there was a positive relationship between obese and blood pressure. Blood pressure and the incidence of hypertension increased with the increase of obese [48]. The prevalence of hypertension was 11.3%, 15.4%, 29.5%, and 44.5% in underweight, normal weight, overweight, and obese groups judged by BMI, respectively [3]. Overweight and obese are important and independent risk factors for the development of hypertension [49], and PA has an impact on blood pressure in people with different obesity status. A Finnish study of the combined effect of obese and PA on hypertension found a protective effect of PA on hypertension risk in both normal-weight and overweight individuals [50]. A Norwegian study showed that high-intensity PA partly reduced the effect of obese on hypertension, especially in men [51]. A study from a long-term cohort of Australian women [52] showed that overweight and obese increased the risk of hypertension by 92% and 252% compared with normal weight women. Both PA and maintaining a healthy weight were beneficial in reducing the risk of hypertension. However, PA could only reduce, but not eliminate the effect of obese on the risk of hypertension. 

The underlying mechanism of the relationship between obese and hypertension is not fully clear [53]. It has been suggested that obese induced an increase in sympathetic nerve activity. While changes in adipokine secretion lead to increased levels of free fatty acids and inflammatory mediators, increased levels of leptin, and decreased levels of adiponectin, which may then lead to impaired insulin signaling in endothelial cells due to the changes in microvascular function. Increased fat could cause hyperinsulinemia, renin-angiotensin-aldosterone system disorder, and lead to vasoconstriction [54,55,56]. Based on the mechanism from previous studies, it was indicated that PA reduced body fat and, therefore, blood pressure.

We found that PA had no direct effect on SBP (Table 2), while BF% played a significant mediation effect on the effect of PA on SBP and DBP, indicating that PA could not directly affect SBP; and it was necessary to reduce SBP through mediation variables, which confirmed the hypothesis of our study. On the other hand, in the model “PA→BF%→DBP”, the total effect was affected by the suppression effect because *ab* and *c’* had the opposite signs; then, it was not statistically significant. In the hypothesis model, the direct effect seemed the main effect due to the absolute value being nearly double than the indirect effect. With the establishment of the “PA→BF%→blood lipids” pathway, we assumed that blood lipids were also the mediator in the effect of PA on blood pressure. Then, we constructed a serial multiple-mediators effect model, “PA→BF%→blood lipids→blood pressure” pathway.

The path “PA→BF%→blood pressure” in path 1 of Table 3 and Table 2 were both established. HDL-C and LDL-C were each mediators of PA on blood pressure due to the paths “PA→HDL-C/LDL-C→SBP” and “PA→LDL-C→DBP”, which were established in path 2 of Table 3. Previous study has pointed out that there were many factors that cause blood pressure elevation, but dyslipidemia was closely related to hypertension, mainly reflected in the excessive intake of dietary saturated fatty acids and a lack of PA, which would lead to increased blood viscosity, thickening of the blood vessel wall, increased vascular endothelial growth factor mobility, resulting in vascular lumen stenosis, slow blood flow speed, and ultimately increasing vascular pressure [57]. HDL-C is the main apolipoprotein in the human body. It has the function of reversing operation, promoting the synthesis of free cholesterol accumulated in peripheral tissues and lipoproteins in the blood circulation, and combining cholesterol with some macromolecules, transporting cholesterol to the liver for metabolism to maintain lipid balance. When adipose tissue increases, the consumption of HDL-C increases, and the accumulation of unmetabolized neutral fat and lipid substances further increases the blood viscosity, leading to dyslipidemia, thereby increasing blood pressure [58]. HDL-C is closely related to blood pressure, but Figure S2A,E showed that the regression coefficient between HDL-C and SBP/DBP was positive, which indicated that SBP/DBP increased with the increase of HDL-C. These results were thought to be contrary to the theoretical interpretation. The results of Yuji team showed that HDL-C level was positively correlated with the incidence of hypertension in people with high levels of circulating CD34-positive cells, but not in people with low levels of circulating CD34-positive cells [11]. With the deepening of cellular and molecular level research, it has been gradually found that the relationship between HDL-C and blood pressure was not static under different conditions. The positive relationship between HDL-C and blood pressure in this study may be reflected by the strong action of some cellular level factors. Therefore, when the relationship between HDL-C and blood pressure is not fully understood, based on the phenomenon found in epidemiology, more mechanistic studies at the cellular and molecular levels are needed to verify it. 

Table 3 also provided the information that most pathways in path 3 were established, but the coefficient values were so small, which indicated a low contribution in PA on blood pressure. However, even a low contribution rate could also explain the effect of intervention at a specific point in the source or pathway to control blood pressure. 

For TC and TG, path 2 was not established, but path 3 was conducted, indicating that the role of TC and TG in the pathway was affected by BF% rather than the direct effect of PA. HDL-C and LDL-C in the model of PA on SBP, and LDL-C in the model of PA on DBP, as the second mediators, were both established in pathway 2 and pathway 3, indicating that PA and BF% have simultaneous and sequential effects on them. It was also possible that there were other mediating variables that have a significant effect on HDL-C and LDL-C and ultimately, achieve the effect of lowering blood pressure. Yang’s results proposed that insulin-related indicators were the mediating variables in the association between BF% and blood pressure [59]. It was concluded that the effects of PA on blood pressure have a specific order, and the pathways of the effect of PA on blood pressure are found from the epidemiological level, which provides a basis for further analysis of the mechanism of how PA affects blood pressure from a cellular and molecular level.

The findings above suggested that blood lipids had an effect on blood pressure after BF%. We also analyzed the pathway “PA→blood lipids→BF%→blood pressure”, but the results were not listed in this paper. However, we found that when HDL-C, LDL-C, and TG were used as the first mediating variables, the pathway was also established, indicating that the effect of BF% and blood lipids was reciprocal, acting together as mediating variables on blood pressure. There might be an interaction between them. Therefore, blood lipids were further assumed to be the moderating variable in the three-segment path of the “PA→BF%→SBP/DBP” mediation model to optimize it. From the moderated mediation model with covariates analysis (Figure 2 and Figure 3), serum lipids were associated with BF% as *a*_2_ were all statistically significant. HDL-C played a moderating role in the latter pathway of the “PA→BF%→SBP/DBP” mediation model, and LDL-C/TC played a moderating role in the direct effect of the “PA→BF%→DBP” in the mediation model (some literature has called the mediated moderated effect [60]). However, TG did not play the moderating role in the “PA→BF%→SBP/DBP” mediation model.

According to the serial multiple-mediation model (Table 3) and the moderated mediation model (Figure 2 and Figure 3), HDL-C not only acted as a second mediator after BF%, but also acted as a moderator together with BF% on blood pressure at the specific position, which reflected the important role of HDL-C in the relationship between BF% and blood pressure.

Although LDL-C has no moderated effect in the “PA→BF%→DBP” mediation model, it acted together with PA on DBP, and LDL-C also played a mediating role as the second mediator in the serial multiple-mediation model, indicating that LDL-C could have acted both as a second mediator or a moderator in the model of PA on DBP. However, LDL-C had an effect on SBP as the second mediator in the mediation model of PA on SBP, but LDL-C did not have a moderated effect in the moderated mediation model. In the serial multiple-mediation model, TC, as the second mediator, did not play a mediating role in path 2, and had little effect in path 3. However, as a moderator, it played a moderating role in the direct effect of PA on DBP. Therefore, although TC played a small role in the model, it also had a certain influence on DBP with the change of PA. TG showed a small effect in the serial multiple-mediation model, and it did not played as a moderator in the “PA→BF%→SBP/DBP” mediation model.

In the serial multiple-mediation model, blood lipids could be used as a mediator, whether it was the first or the second mediator, and they were all statistically significant in the models. We observed that the effect on BF% was statistically significant when blood lipids were used as the first mediator. However, in the moderated mediation model (Figure 2 and Figure 3), blood lipids had no moderated effect in the anterior path “PA→BF%”, but had moderated effect in the posterior path “BF%→SBP/DBP”. The results showed that blood lipids and PA did not interact with BF%, but only acted as the influencing factor of BF%. This effect was similar to that of blood lipids as the first mediator in the serial multiple-mediation model. Meanwhile, blood lipids interacted with BF% and affected blood pressure. Therefore, we believed that among the three models that have been constructed, serum lipids played as the moderator; and the moderated mediation model was more appropriate to analyze the association among PA, BF%, blood lipids, and blood pressure.

The physiological mechanism could be inferred from our moderated mediation models. According to our previous results and references, we know that PA did not affect the blood pressure directly. The lack of PA increased the BF%; and adipose tissue would release the negative adipokines like leptin, resistin, and chemoattractant, which were the “bad adipokines” promoting atherosclerosis and endothelial dysfunction [47]. When adipose tissue increases, the consumption of HDL-C increases, and the accumulation of unmetabolized neutral fat and lipid substances further increases the blood viscosity, leading to dyslipidemia, thereby increasing blood pressure [58]. Then, these would lead to increased blood viscosity, thickening of the blood vessel wall, increased vascular endothelial growth factor mobility, resulting in vascular lumen stenosis, slow blood flow speed, and ultimately increase vascular pressure [57]. However, further experiments at the molecular level are needed to test our hypothesis.

Our study extends contemporary knowledge in several important ways. First, based on the mediation effect model, our study is the first study discussing the association between PA and CVD risk factors at the same time in China. This study only preliminarily explored the order relationship and influence mode of PA, BF%, and CVD risk factors from the epidemiological level, and found the connecting role of blood lipids in the pathway. This is an exploration of mechanisms of epidemiology to solve the “black box” problem. We also tried to analyze the “PA→BF%→blood pressure→blood lipids” pathway, because it has been reported that the effects of blood lipids and blood pressure were interactive. But, the sample data of this study showed that the “blood pressure→blood lipids” pathway was not established, and the results were not listed in this paper. This study was based on the hypothesis that the relationship between PA and CVD was statistically significant, the interaction between CVD was known from the references, and the results were verified by statistical analysis. However, the analysis of mediation variables might be insufficient. Also, in the models, we adjusted for as many confounders as possible, including social demographic characteristics, diet, and physical measurement; and the estimated values were reasonable and strictly controlled. When a large number of confounders were controlled, the stability of the effects of independent variables, dependent variables, and mediating variables depends on the sample size. CHNS data structure was based on hierarchical sampling scheme, which required the application of a multilevel mediation effect model. In order to satisfy both the sample size and the multilevel data structure, the further research is to use data from multiple survey rounds for analysis. 

Some limitations of this study should be considered. First, though CHNS was a long-term follow-up study, 2015 was the first year to collect body composition data. But, the public data of BF% were only in 2015. Second, the anthropometric methods we used to measure PA and BF% in our study were not the most precise available, but they were broadly acceptable and were the most economical and useful way for a large-scale survey covering fifteen provinces in China. PA questionnaire was effective and validated by professors from the University of North Carolina at Chapel Hill, as well as the National Institute for Nutrition and Health, Chinese Center for Disease Control and Prevention. BF% was measured by bioelectrical impedance analysis because it was cost-efficient and easily carried. Third, a large proportion of the participants were excluded from our analysis for varying reasons, which meant the generalization of our results to the entire survey population should only be made with caution. Lastly, the mediation models in our study were based on the previous basic theory, and many mediators and confounders might not be taken into account. 

In a further study, we could consider more variables when we would collect more data, and construct more reasonable models like a multiple-level moderated mediation effect model to reveal more robust associations between PA and CVD risk factors and figure out the complex “black box” question.

## 5. Conclusions

The findings from the present study indicate that BF% played a mediating role in the relationship between PA and blood pressure. HDL-C, LDL-C, and TC were more likely to act as moderators in the mediation model “PA→BF%→SBP/DBP”, while TG had little effect in the model. PA could directly and indirectly benefit to control the CVD risk factors simultaneously. 

## Figures and Tables

**Table 1:** Demographic characteristics of samples from fifteen provinces of China in 2015.

Characteristics	Male (*n* = 4647)	Female (*n* = 5501)	Total (*n* = 10,148)
Age (years old)	50.95 (40.70, 61.16)	50.08 (39.82, 60.38)	50.57 (40.19, 60.73)
Education (%)			
High school and above (%)	1841 (39.62)	1826 (33.19)	3667 (36.14)
Junior school (%)	1628 (35.03)	1697 (30.85)	3325 (32.77)
Primary school and illiteracy (%)	1178 (25.35)	1978 (35.96)	3156 (31.10)
Married (%)	4177 (89.89)	4825 (87.71)	9002 (88.71)
Annual per capita household income (/1000 yuan)	16.5 (7.5, 28.9)	15.7 (6.9, 28.2)	16.1 (7.2, 28.6)
Current smoker (%)	2355 (50.68)	87 (1.58)	2442 (24.06)
Alcohol drinker (%)	2544 (54.74)	363 (6.60)	2907 (28.65)
PA (MET·h/week)	110.00 (33.40, 217.58)	107.45 (48.30, 202.20)	108.27 (42.48, 209.92)
Sedentary activity time (h/week)	17.50 (11.50, 30.00)	15.50 (9.33, 28.00)	16.00 (10.50, 28.00)
Energy intake (1000 kcal/day)	2.14 (1.71, 2.66)	1.77 (1.42, 2.21)	1.93 (1.53, 2.43)
Energy from dietary fat (%)	35.24 (27.25, 43.54)	35.43 (27.61, 43.54)	35.34 (27.43, 43.54)
Dietary cholesterol (mg/d)	226.44 (112.75, 374.03)	194.61 (92.76, 324.46)	207.21 (101.48, 346.50)
Dietary sodium-to-potassium ratio	2.71 (1.78, 3.98)	2.65 (1.73, 3.96)	2.67 (1.75, 3.97)
BMI (kg/m^2^)	23.88 (21.57, 26.35)	23.49 (21.32, 25.90)	23.67 (21.41, 26.12)
Waist circumference (cm)	86.00 (79.00, 93.00)	81.00 (74.00, 88.00)	83.20 (76.00, 90.40)
BF% (%)	22.60 (18.10, 26.50)	33.40 (28.70, 37.80)	28.00 (22.10, 34.60)
SBP (mm Hg)	125.33 (116.67, 137.33)	120.00 (110.00, 132.00)	122.00 (112.33, 134.67)
DBP (mm Hg)	80.67 (76.00, 88.66)	78.67 (71.00, 83.33)	80.00 (72.67, 86.00)
HDL-C (mmol/L)	1.18 (0.99, 1.41)	1.30 (1.10, 1.52)	1.25 (1.05, 1.48)
LDL-C (mmol/L)	3.06 (2.51, 3.63)	2.99 (2.43, 3.60)	3.02 (2.47, 3.62)
TG(mmol/L)	1.26 (0.85, 1.98)	1.11 (0.77, 1.64)	1.18 (0.80, 1.78)
TC (mmol/L)	4.80 (4.19, 5.44)	4.85 (4.20, 5.50)	4.83 (4.20, 5.48)

Education, marital status, current smoker, and alcohol consumer were expressed as a number (proportion). Age, annual per capita household income, sedentary activity time, energy intake, energy from dietary fat, dietary cholesterol, dietary sodium-to-potassium ratio, body mass index (BMI), waist circumference, physical activity (PA), body-fat percentage (BF%), systolic blood pressure (SBP), diastolic blood pressure (DBP), high-density lipoprotein cholesterol (HDL-C), low-density lipoprotein cholesterol (LDL-C), total cholesterol (TC), and triglyceride (TG) were expressed as median (25th, 75th) because of their abnormal distribution.

**Table 2:** The coefficients (95% CI) of the simple mediation models between physical activity and blood pressure, serum lipids among Chinese adults from fifteen provinces in 2015.

Dependent Variable	Direct Effect *c*′	Indirect Effect *ab*	Total Effect *c = ab + c*′	*ab/c*
SBP	0.0556 (−0.2208, 0.3321)	−0.1491 (−0.2030, −0.0991) *	−0.0935 (−0.3736, 0.1865)	-
DBP	0.2328 (0.0546, 0.4109) *	−0.1120 (−0.1508, −0.0754) *	0.1208 (−0.0606, 0.3022)	0.48 ^#^
HDL-C	0.0177 (0.0117, 0.0238) *	0.0052 (0.0036, 0.0069) *	0.0229 (0.0167, 0.0292) *	0.23
LDL-C	−0.0287 (−0.0451, −0.0124) *	−0.0097 (−0.0130, −0.0067) *	−0.0384 (−0.0550, −0.0219) *	0.25
TC	−0.0117 (−0.0312, 0.0077)	−0.0067 (−0.0092, −0.0043) *	−0.0184 (−0.0380, 0.0011)	-
TG	−0.0167 (−0.0387, 0.0053)	−0.0151 (−0.0201, −0.0103) *	−0.0317 (−0.0542, −0.0093) *	-

HDL-C, high-density lipoprotein cholesterol; LDL-C, low-density lipoprotein cholesterol; TC, total cholesterol; TG, hypertriglyceridemia; SBP, systolic blood pressure; DBP, diastolic blood pressure. All the models were constructed using simple mediation models. Models adjusted sex, age, current smoker, alcohol drinker, per capita household income, sedentary activity time, energy intake, energy for dietary fat, dietary cholesterol (only for blood lipids), dietary sodium-to-potassium ratio (only for SBP and DBP). ^#^ When both direct effect and indirect effect are statistically significant and the symbol is opposite, we report |*ab/c*′|. * *p* < 0.05.

**Table 3:** The coefficients (95% CI) of the serial multiple-mediator models between physical activity and blood pressure mediated by serum lipids among Chinese adults in fifteen provinces in 2015.

*Y*	*W*	Total Effect	Direct Effect *c*′	Mediation Effect			
				*a*_1_*b*_1_ + *a*_2_*b*_2_ + *a*_1_*a*_3_*b*_2_	Path 1 (*a*_1_*b*_1_)	Path 2 (*a*_2_*b*_2_)	Path 3 (*a*_1_*a*_3_*b*_2_)
SBP	HDL-C	−0.1700 (−0.4698, 0.1298)	−0.0225 (−0.3188, 0.2738)	−0.1475 (−0.2068, −0.0887) *	−0.1798 (−0.2398, −0.1206) *	0.0250 (0.0065, 0.0480) *	0.0073 (0.0019, 0.0140) *
SBP	LDL-C	−0.1684 (−0.4681, 0.1313)	0.0422 (−0.2530, 0.3373)	−0.2105 (−0.2729, −0.1519) *	−0.1598 (−0.2142, −0.1085) *	−0.0380 (−0.0638, −0.0155) *	−0.0127 (−0.0189, −0.0078) *
SBP	TC	−0.1665 (−0.4662, 0.1332)	0.0211 (−0.2738, 0.3160)	−0.1876 (−0.2516, −0.1262) *	−0.1652 (−0.2203, −0.1124) *	−0.0143 (−0.0393, 0.0102)	−0.0081 (−0.0122, −0.0049) *
SBP	TG	−0.1652 (−0.4649, 0.1346)	0.0191 (−0.2763, 0.3145)	−0.1843 (−0.2456, −0.1270) *	−0.1603 (−0.2149, −0.1085) *	−0.0125 (−0.0315, 0.0046)	−0.0114 (−0.0174, −0.0066) *
DBP	HDL-C	0.0328 (−0.1617, 0.2272)	0.1586 (−0.0327, 0.3499)	−0.1258 (−0.1699, −0.0837) *	−0.1288 (−0.1717, −0.0881) *	0.0023 (−0.0096, 0.0146)	0.0007 (−0.0029, 0.0043)
DBP	LDL-C	0.0301 (−0.1643, 0.2244)	0.1808 (−0.0098, 0.3714)	−0.1507 (−0.1960, −0.1083) *	−0.1206 (−0.1599, −0.0835) *	−0.0226 (−0.0381, −0.0090) *	−0.0076 (−0.0114, −0.0046) *
DBP	TC	0.0313 (−0.1631, 0.2257)	0.1686 (−0.0218, 0.3589)	−0.1373 (−0.1817, −0.0945) *	−0.1239 (−0.1650, −0.0855) *	−0.0085 (−0.0236, 0.0063)	−0.0048 (−0.0073, −0.0028) *
DBP	TG	0.0348 (−0.1596, 0.2291)	0.1737 (−0.0166, 0.3640)	−0.1389 (−0.1856, −0.0962) *	−0.1177 (−0.1585, −0.0800) *	−0.0111 (−0.0275, 0.0040)	−0.0101 (−0.0150, −0.0063) *

HDL-C, high-density lipoprotein cholesterol; LDL-C, low-density lipoprotein cholesterol; TC, total cholesterol; TG, hypertriglyceridemia; SBP, systolic blood pressure; DBP, diastolic blood pressure. Mediator *M* was body-fat percentage and *W* represents the second mediator. All the models were constructed using serial multiple-mediator models. Models adjusted sex, age, current smoker, alcohol drinker, per capita household income, sedentary activity time, energy intake, energy for dietary fat, dietary cholesterol, and dietary sodium-to-potassium ratio. * *p* < 0.05.

## Data Availability

The datasets generated during and/or analyzed during the present study are available from the author (B.Z.) upon reasonable request.

## Supplementary Materials

The following supporting information can be downloaded at: https://www.mdpi.com/article/10.3390/nu15143113/s1, Figure S1: Diagram of the simple mediation models between physical activity and blood pressure, blood lipides among Chinese adults in fifteen provinces in 2015. The mediator is body fat%. The arrows indicate the direction. The solid line indicates that the coefficient is significant and the pathway is established. The dashed line indicates that the coefficient is not significant and the pathway is not valid. Omit the covariates and error terms. * *p* < 0.05.; Figure S2: Diagram of the serial multiple mediator models between physical activity and blood pressure among Chinese adults from 15 provinces in 2015. The first mediator is the body fat%. The second mediator is HDL-C/LDL-C. The arrows indicate the direction. The solid line indicates that the coefficient is significant and the pathway is established. The dashed line indicates that the coefficient is not significant and the pathway is not valid. Omit the covariates and error terms. * *p* < 0.05.
