# Supplementary material for: The Mediation and Moderation Effect Association among Physical Activity, Body-Fat Percentage, Blood Pressure, and Serum Lipids among Chinese Adults: Findings from the China Health and Nutrition Surveys in 2015
Source: Nutrients. 2023 Jul 12;15(14):3113. doi: 10.3390/nu15143113 (PMC10383535; doi:10.3390/nu15143113)
Supplement: Supplementary file 1 [file nutrients-15-03113-s001.zip › Figure S1.pdf]

nutrients

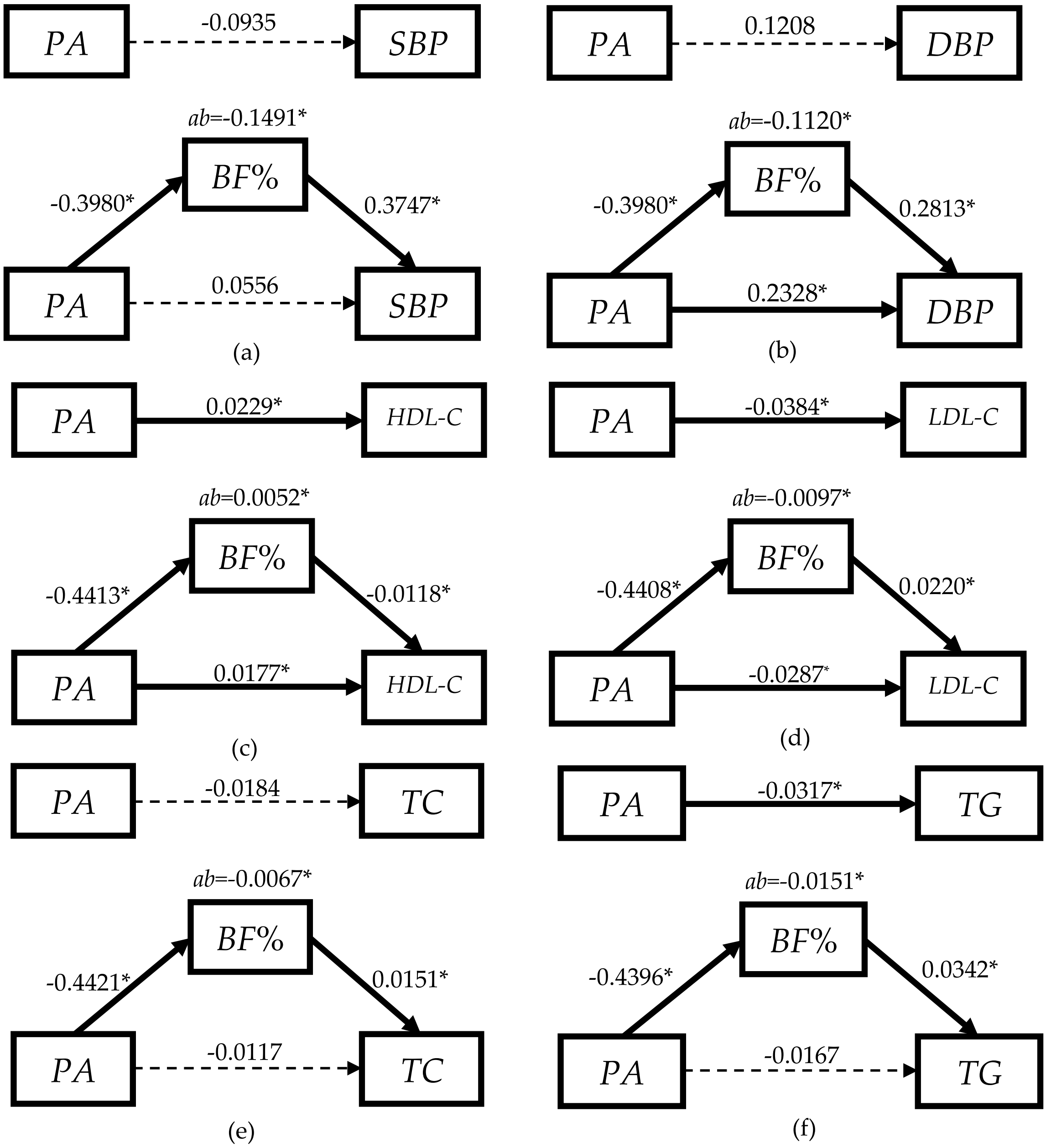


**Figure S1.** Diagram of the simple mediation models between physical activity and blood pressure, blood lipides among Chinese adults in fifteen provinces in 2015. The mediator is body fat%. The arrows indicate the direction. The solid line indicates that the coefficient is significant and the pathway is established. The dashed line indicates that the coefficient is not significant and the pathway is not valid. Omit the covariates and error terms. * $p < 0.05$.
