# Supplementary material for: The Mediation and Moderation Effect Association among Physical Activity, Body-Fat Percentage, Blood Pressure, and Serum Lipids among Chinese Adults: Findings from the China Health and Nutrition Surveys in 2015
Source: Nutrients. 2023 Jul 12;15(14):3113. doi: 10.3390/nu15143113 (PMC10383535; doi:10.3390/nu15143113)
Supplement: Supplementary file 1 [file nutrients-15-03113-s001.zip › Figure S2.pdf]

nutrients

A. PA→BF%→HDL-C→SBP, mediator *M* is BF% and mediator *W* is HDL-C.

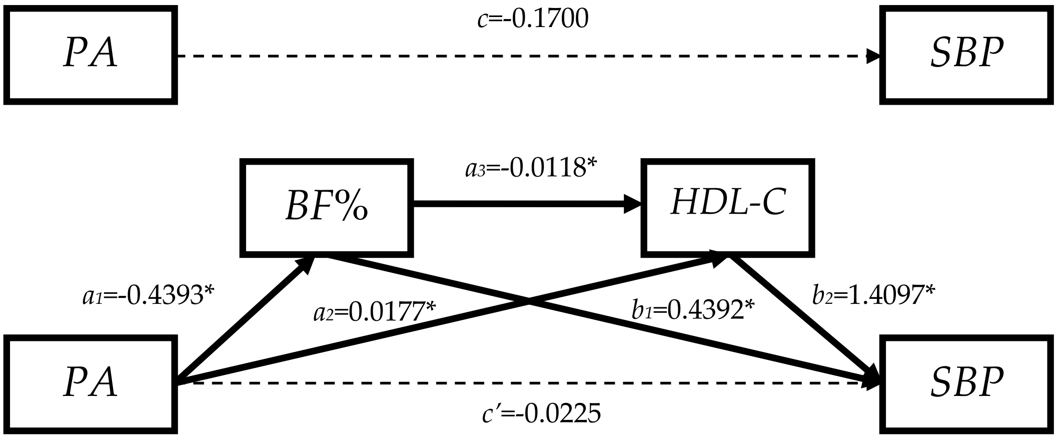

B. PA→BF%→LDL-C→SBP, mediator *M* is BF% and mediator *W* is LDL-C.

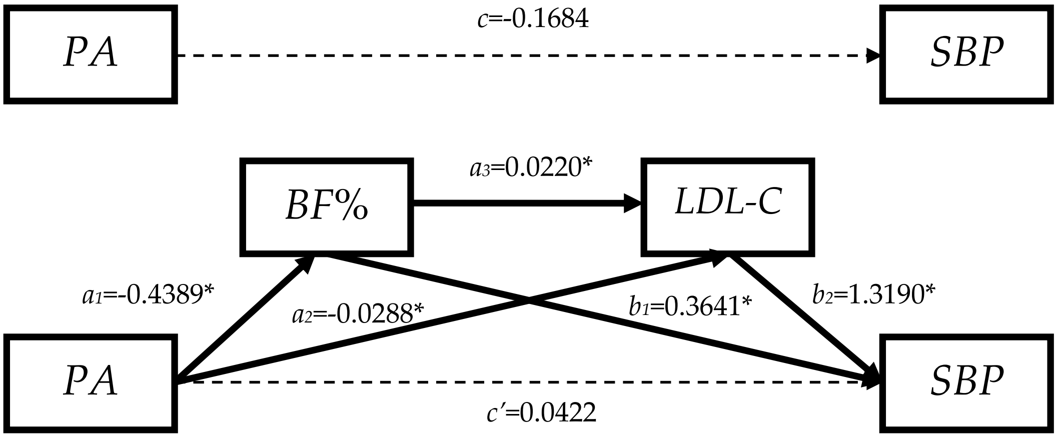

C. PA→BF%→TC→SBP, mediator *M* is BF% and mediator *W* is TC.

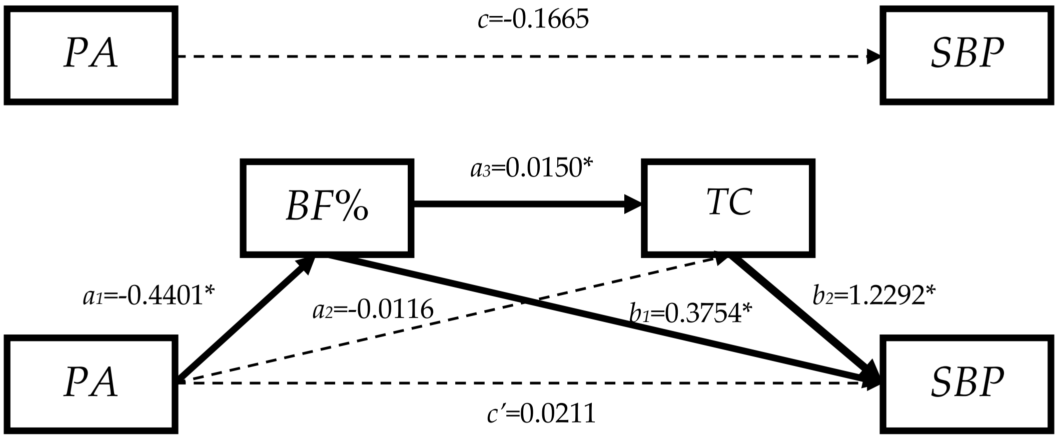

nutrients MDPI

D. PA→BF%→TG→SBP, mediator *M* is BF% and mediator *W* is TG.

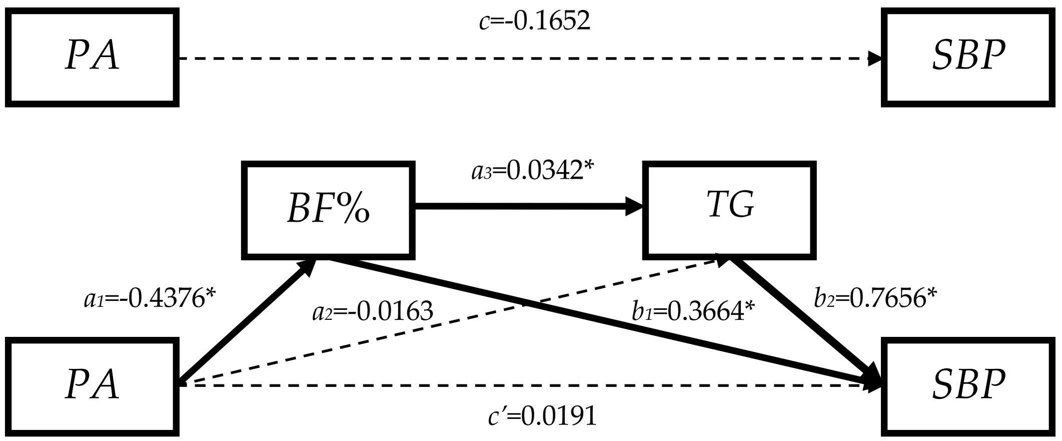

E. PA→BF%→HDL-C→DBP, mediator *M* is BF% and mediator *W* is HDL-C.

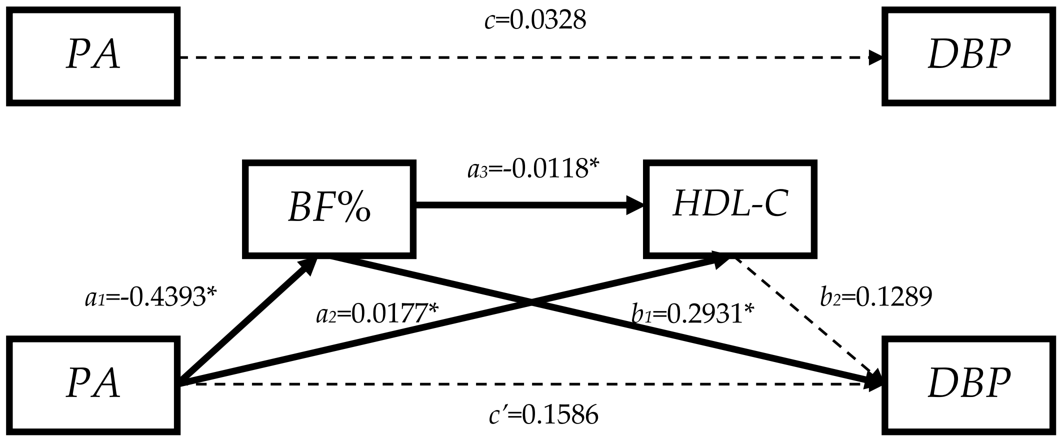

F. PA→BF%→LDL-C→DBP, mediator *M* is BF% and mediator *W* is LDL-C.

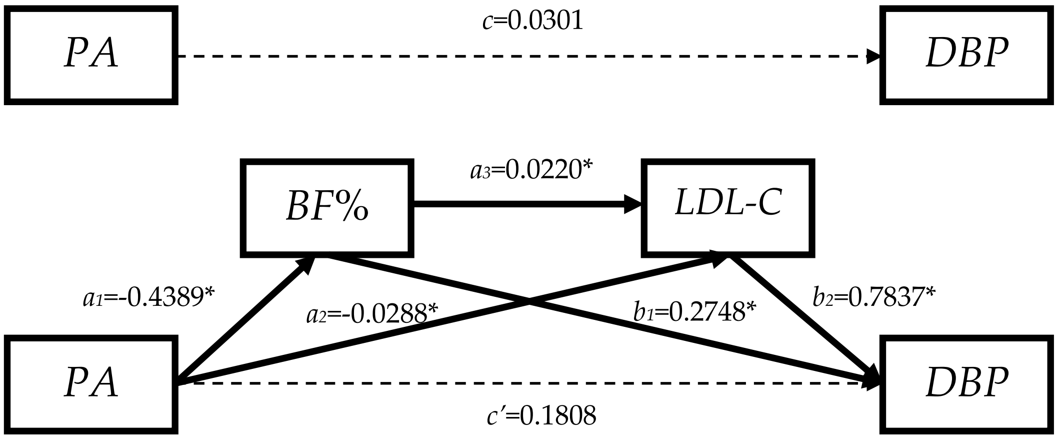

G. PA→BF%→TC→DBP, mediator *M* is BF% and mediator *W* is TC.

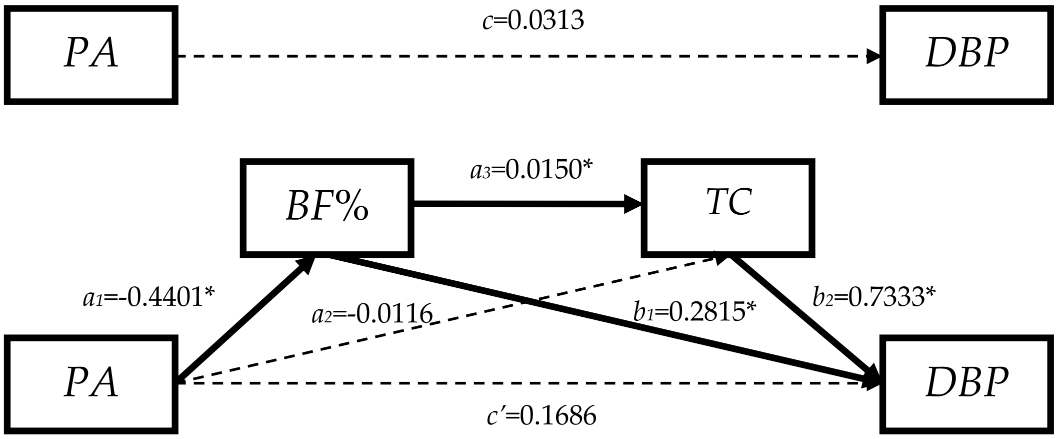

H. PA→BF%→TG→DBP, mediator *M* is BF% and mediator *W* is TG.

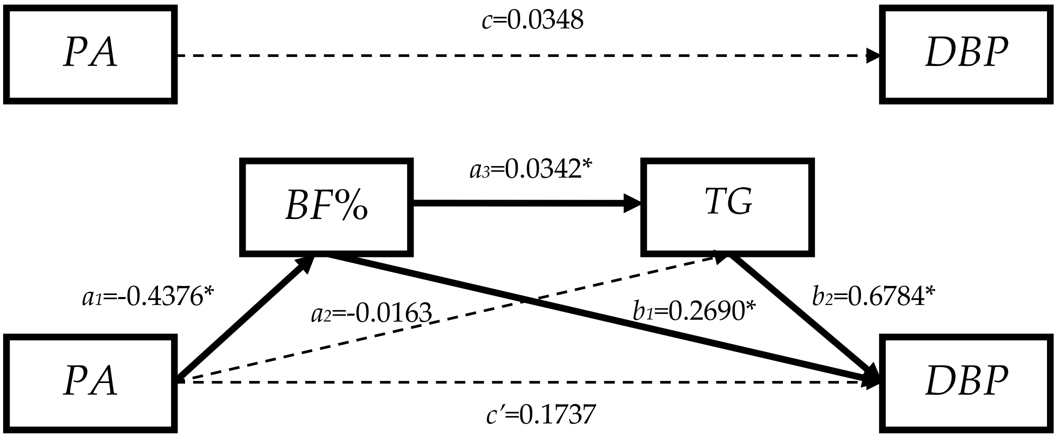

nutrients

MDPI

Figure S2. Diagram of the serial multiple mediator models between physical activity and blood pressure among Chinese adults from 15 provinces in 2015. The first mediator is the body fat%. The second mediator is HDL-C/LDL-C. The arrows indicate the direction. The solid line indicates that the coefficient is significant and the pathway is established. The dashed line indicates that the coefficient is not significant and the pathway is not valid. Omit the covariates and error terms. * $p < 0.05$.
